# Supplementary material for: How Can Ozone and Relative Humidity Affect Artists’ Alkyd Paints? A FT-IR and Py-GC/MS Systematic Study
Source: Polymers (Basel). 2022 Apr 29;14(9):1831. doi: 10.3390/polym14091831 (PMC9101010; doi:10.3390/polym14091831)
Supplement: Supplementary file 1 [file polymers-14-01831-s001.zip › polymers-1701449-supplementary.pdf]

SUPPLEMENTARY MATERIAL

How can ozone and relative humidity affect artists' alkyd paints? A FT-IR and Py-GC/MS systematic study

Laura Pagnin[1], Elisabetta Zendri[2], Francesca Caterina Izzo[2*]

[1] *Academy of Fine Arts Vienna, Institute of Science and Technology in Art, Schillerplatz 3, 1010 Vienna, Austria*

[2] *Ca' Foscari University of Venice, Department of Environmental Sciences, Informatics and Statistics, Via Torino 155/b, 30174 Venice, Italy*

*Email: fra.izzo@unive.it

Table of Contents

Figure S1. TIC (total ion current) pyrogram obtained after Py-GC/MS analysis of pure alkyd resin (Alk_ref), with PY37 (Alk_ref_PY37) and PB29 (Alk_ref_PB29) zoomed in the phthalic acid area (8.71 min); mass spectrum of a lactone-similar structure highlighted in PY37 and PB29 right after phthalic acid. 2

Figure S2. ATR-FTIR spectra of alkyd binder mixed with a) PW6, b) PY37, c) PB29, d) PR101. Comparison between unaged (black), O_3 + 50% RH (red), and O_3 + 80% RH (blue) aged samples. 3

Figure S3. TIC (total ion current) pyrogram obtained after Py-GC/MS analysis of alkyd resin with PR101 before (Alk_PR101_ref) and after ozone ageing at 80%RH (Alk_PR101_80%RHO3) zoomed in the benzoic area (5.71 min). 4

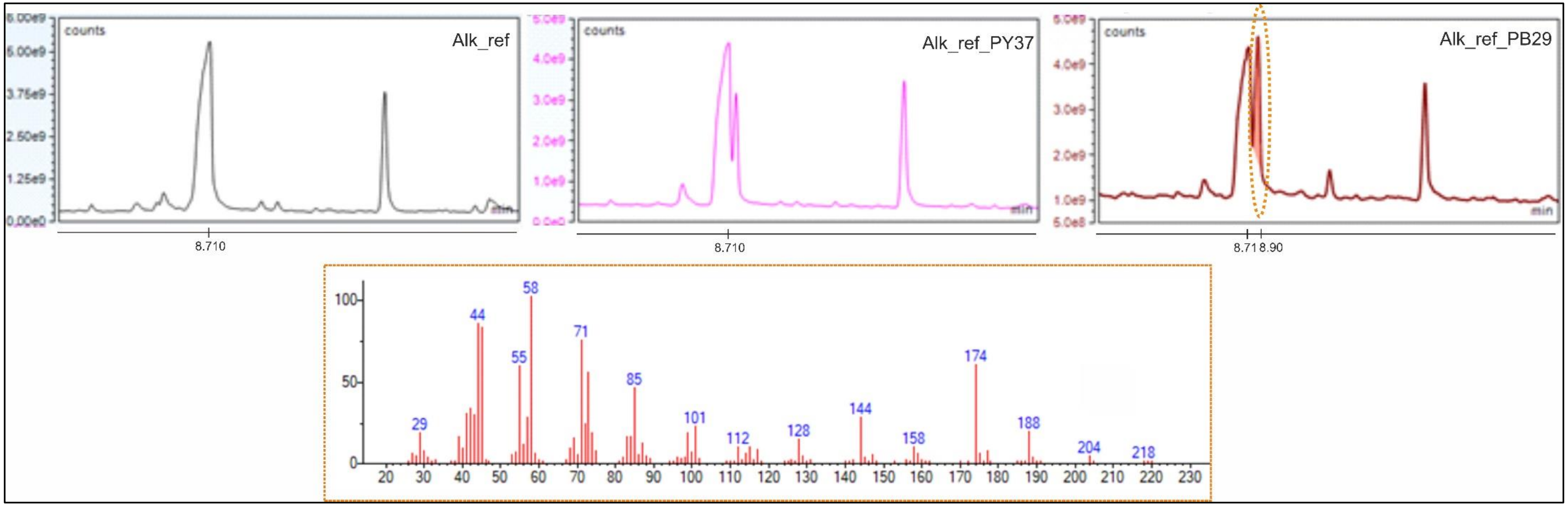

Figure S1. TIC (total ion current) pyrogram obtained after Py-GC/MS analysis of pure alkyd resin (Alk_ref), with PY37 (Alk_ref_PY37) and PB29 (Alk_ref_PB29) zoomed in the phthalic acid area (8.71 min); mass spectrum of a lactone-similar structure highlighted in PY37 and PB29 right after phthalic acid.

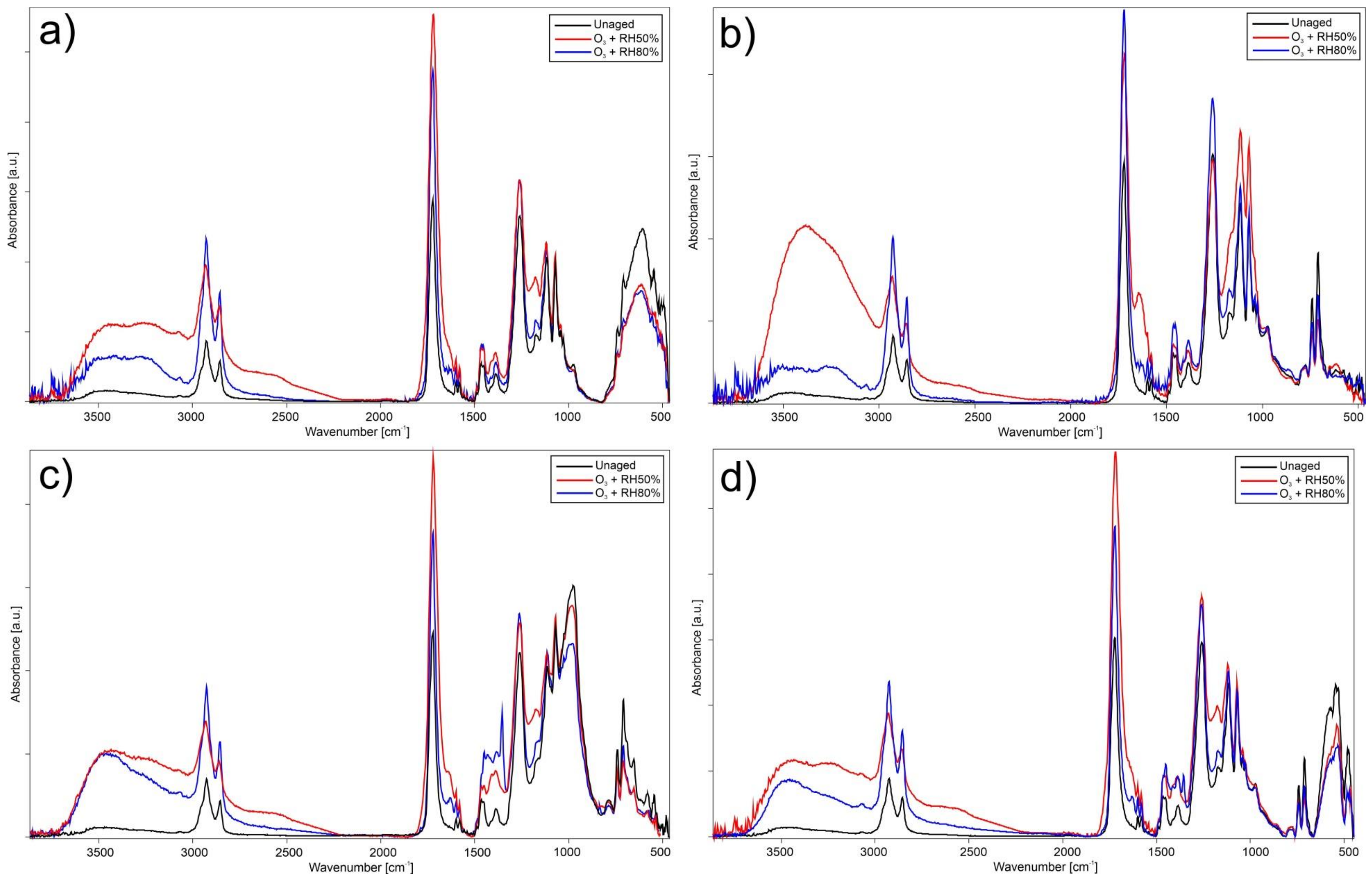

Figure S2. ATR-FTIR spectra of alkyd binder mixed with a) PW6, b) PY37, c) PB29, d) PR101. Comparison between reference (black), O_3 + 50% RH (red), and O_3 + 80% RH (blue) aged samples.

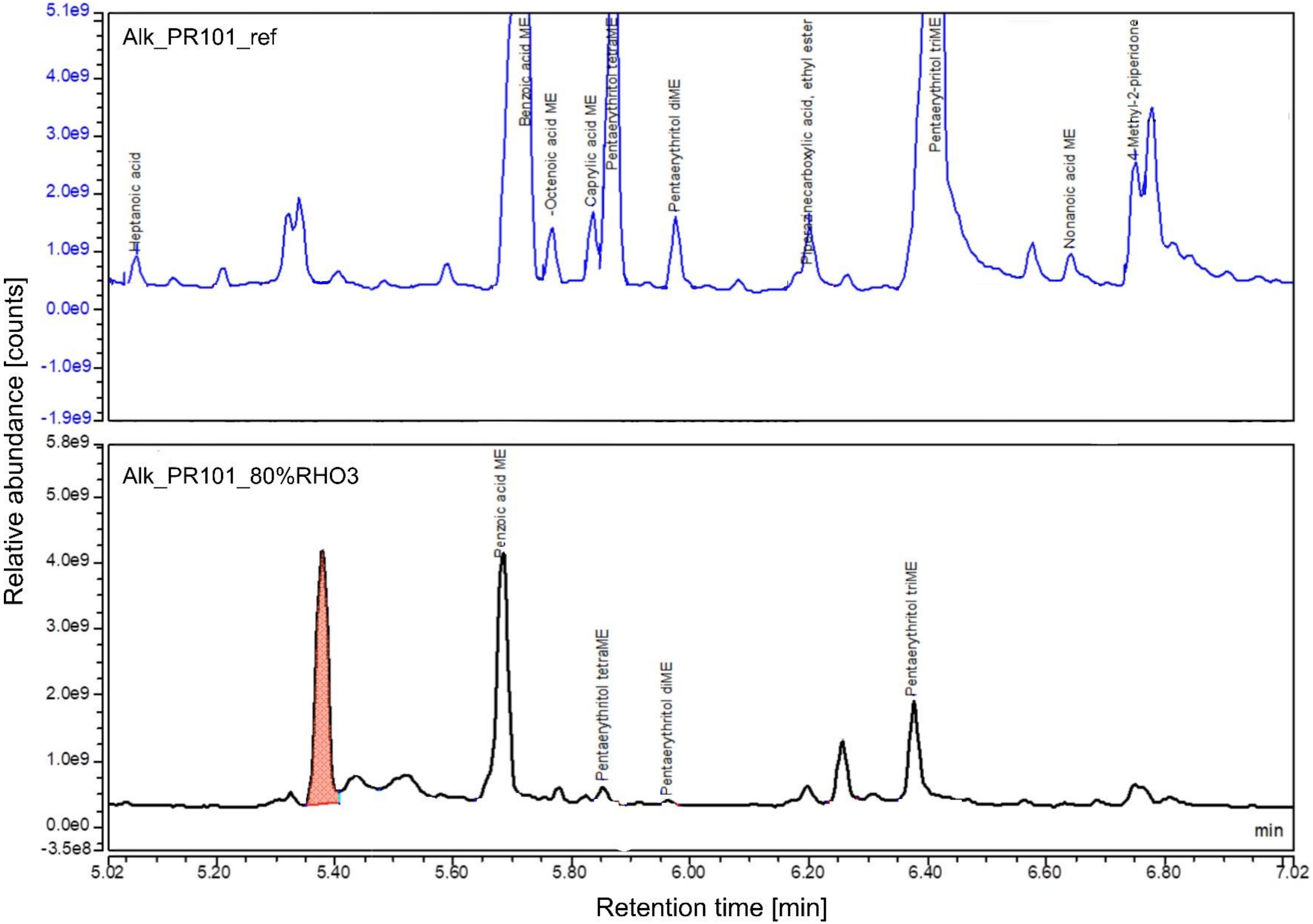

Figure S3. TIC (total ion current) pyrogram obtained after Py-GC/MS analysis of alkyd resin with PR101 before (Alk_PR101_ref) and after ozone ageing at 80%RH (Alk_PR101_80%RHO3) zoomed in the benzoic area (5.71 min).
